# Canonical Notch signaling plays an instructive role in auditory supporting cell development

**DOI:** 10.1038/srep19484

**Published:** 2016-01-20

**Authors:** Dean P. Campbell, Elena Chrysostomou, Angelika Doetzlhofer

**Affiliations:** 1 Solomon H. Snyder Department of Neuroscience, Johns Hopkins University, School of Medicine Baltimore, MD 21205, USA; 2 Center for Sensory Biology, Johns Hopkins University, School of Medicine Baltimore, MD 21205, USA

## Abstract

The auditory sensory epithelium, composed of mechano-sensory hair cells (HCs) and highly specialized glial-like supporting cells (SCs), is critical for our ability to detect sound. SCs provide structural and functional support to HCs and play an essential role in cochlear development, homeostasis and repair. Despite their importance, however, surprisingly little is known about the molecular mechanisms guiding SC differentiation. Here, we provide evidence that in addition to its well-characterized inhibitory function, canonical Notch signaling plays a positive, instructive role in the differentiation of SCs. Using γ-secretase inhibitor DAPT to acutely block canonical Notch signaling, we identified a cohort of Notch-regulated SC-specific genes, with diverse functions in cell signaling, cell differentiation, neuronal innervation and synaptogenesis. We validated the newly identified Notch-regulated genes *in vivo* using genetic gain (*Emx2*^*Cre*/+^; *Rosa26*^*N1ICD*/+^) and loss-of-function approaches *(Emx2*^*Cre*/+^; *Rosa26*^*DnMAML1*/+^). Furthermore, we demonstrate that Notch over-activation in the differentiating murine cochlea (*Emx2*^*Cre*/+^; *Rosa26*^*N1ICD*/+^) actively promotes a SC-specific gene expression program. Finally, we show that outer SCs –so called Deiters’ cells are selectively lost by prolonged reduction (*Emx2*^*Cre*/+^; *Rosa26*^*DnMAML1*/+/+^) or abolishment of canonical Notch signaling (*Fgfr3-iCreER; Rbpj*^*−*/Δ^), indicating a critical role for Notch signaling in Deiters’ cell development.

The auditory sensory organ, housed in the inner ear cochlea, is critical for our ability to hear. It contains a highly specialized sensory epithelium composed of rows of mechano-receptor cells so called inner and outer hair cells (HCs), surrounded by glia-like supporting cells (SCs). Based on morphology, location, and function, SCs are categorized into five sub-types. Border cells and inner phalangeal cells surround inner HCs, inner and outer pillar cells separate inner from outer HCs, and Deiters’ cells surround outer HCs. During cochlear development, SCs provide essential cues that direct the cellular patterning, planar cell polarity as well as innervation and synaptogenesis of HCs. In the mature cochlea, SCs provide structural support and are essential for the survival and proper function of HCs and innervating neurons^1^,^2^. SCs and their neighboring HCs originate from a common pool of post-mitotic progenitors, referred to as pro-sensory cells^3^. Shortly after all pro-sensory cells have withdrawn from the cell cycle, an event, that in mice occurs at around E13.5, up-regulation of the transcription factor ATOH1 in a subset of basally located pro-sensory cells initiates a basal-to-apical wave of HC differentiation^4^. SC differentiation closely follows HC differentiation and by E18.5 the entire length of the sensory epithelium is patterned into a mosaic of intercalating HCs and SCs. The molecular signals/factors that induce SC differentiation are poorly understood. Interestingly, ectopic generation of HCs stimulates the formation of surrounding SC-like cells, suggesting that HCs provide cue(s) that instruct SC differentiation^5^. A candidate for such instructive signal is the Notch signaling pathway.

Canonical Notch signaling is activated when a membrane-bound Notch ligand (JAGGED 1, 2 and DELTA-LIKE 1, 3, 4) interacts with a single-pass transmembrane Notch receptor (NOTCH 1–4), expressed on a neighboring cell. Ligand binding initiates the sequential proteolytic cleavage of the Notch receptor freeing its intracellular domain (NICD) to translocate into the nucleus. In the nucleus, the NICD forms a complex with the transcription factor RBP-J and a member of the Mastermind-Like (MAML) co-factor family activating the transcription of Notch target genes^6^. In the developing inner ear, Notch signaling has been extensively studied for its early, instructive role in pro-sensory development and its inhibitory role in HC fate determination. A critical component of the early function(s) of Notch signaling is the Notch ligand JAGGED1 (JAG1). *Jag1*, a direct Notch target itself, is highly expressed in pro-sensory cells and later becomes confined to SCs^7^. Early otic ablation of *Jag1* results in loss of vestibular sensory structures and abnormal auditory sensory cell development, in which inner HCs are over-produced and outer HCs are largely missing^8^,^9^. The Notch receptor(s) that mediate JAG1 functions in early vestibular and auditory development have not yet been identified. The Notch receptor NOTCH1 and the HC-specific Notch ligands DELTA-LIKE 1 (DLL1) and JAGGED2 (JAG2) are critical components of a later, inhibitory function of Notch signaling in HC fate determination^8^,^10^. Co-deletion of *Dll1* and *Jag2* or deletion of *Notch1* results in massive HC over-production at the cost of SCs^11^. The HC-repressive function of Notch signaling is thought to be mediated by members of the HES/HEY family of transcriptional repressors. HES/HEY factors are known to antagonize the HC fate promoting activity of ATOH1^12^,^13^ and deletion of *Hes/Hey* genes results in an overproduction of HCs^12^,^14^,^15^,^16^.

Here, we provide evidence that Notch signaling not only suppresses a HC fate in pro-sensory cells, but instructs their development as SCs. We identify SC-specific Notch-regulated genes with functions in cell-cell signaling, neuronal innervation and glial physiology. We show that Notch signaling is sufficient to ectopically induce a SC-specific gene expression program, and is sufficient to render outer HC precursors and a subset of non-sensory epithelial cells into SC-like cells. Finally, we demonstrate that disruption of canonical Notch signaling in the differentiating cochlea results in the selective death of differentiating Deiters’ cells, revealing a critical role for Notch signaling in Deiters’ cell development.

## Results

### Identification of Notch-regulated genes in the differentiating cochlea

**Figure 1:**
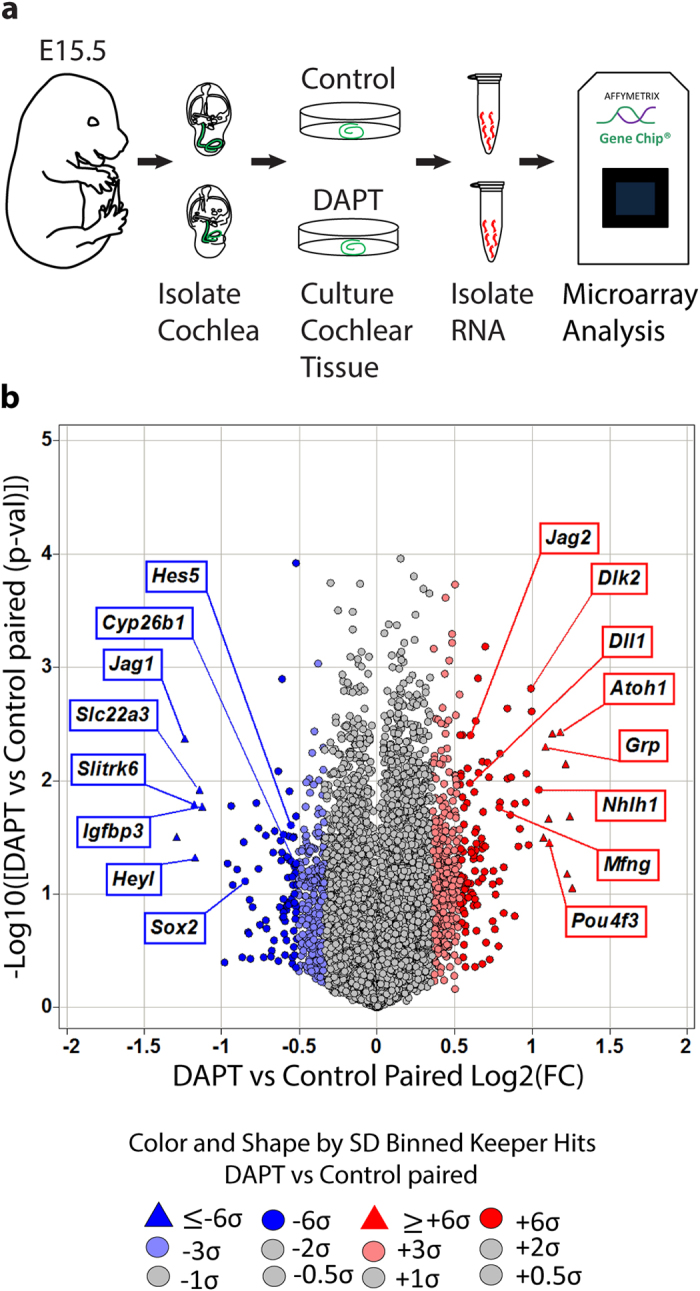
Identification of Notch-regulated genes in the differentiating cochlea. (**a**) Schematics of experimental approach used to uncover novel Notch-regulated transcripts. Transcript changes in E15.5 cochlear epithelial cells after ~20 hours of DMSO (control) or GSI (DAPT) treatment were analyzed using GeneChip® Mouse Exon 1.0 ST Arrays. **(b)** Volcano plot of microarray data. Plotted is log2 fold-change (x-axis) versus −log10 p-value (y-axis). Note that transcripts that are significantly up-regulated in response to DAPT treatment are marked in dark red circles (log2 (FC) > 3σ) and triangles (log2 (FC) > 6σ); transcripts that are significantly down-regulated in response to DAPT treatment are marked in dark blue circles (log2 (FC) < −3σ) and triangles (log2 (FC) < −6σ). Abbreviations: fold change (FC), standard deviation (SD).

To gain insights into the function(s) of Notch signaling in differentiating SCs, we characterized the transcriptional targets of Notch signaling in the differentiating cochlea. To block Notch signaling we used DAPT, a γ-secretase inhibitor (GSI), known to efficiently block Notch receptor cleavage in intact cells^17^. We cultured wild type cochlear tissue at E15.5 in the presence of GSI DAPT or vehicle control DMSO (control) for 19–22 hours. At the end of the culture period, we pooled control and DAPT treated explants, enzymatically purified the cochlear epithelial duct, and extracted RNA. Control and DAPT treated RNA samples from three independent experiments were analyzed using the GeneChip® Mouse Exon ST Arrays (Fig. 1a). Using a one-way ANOVA-model we determined genes that were significantly changed in control versus DAPT treated cochlear epithelial cells (Fig. 1b). Consistent with having disrupted the HC-repressive function of Notch signaling, HC-specific transcription factors (e.g. *Atoh1*^18^, *Pou4f3*^19^ and *Nhlh1*^20^) and HC-specific Notch signaling components (*Dll1*^7^, *Jag2*^10^ *Mfng*^21^ were significantly up-regulated (fold change (FC) ≥ +6σ, p-value ≤ 0.05) in response to DAPT treatment (Fig. 1b, red) (Supplementary Table S1). Conversely, known Notch target genes involved in HC fate repression (e.g. *Hey1*^16^, *HeyL*^13^) and pro-sensory development (e.g. *Jag1*^22^, *Sox2*^23^ were among the genes that were significantly down-regulated (FC ≤ −6σ, p-value ≤ 0.05) in response to DAPT. However, a large fraction of the genes downregulated in response to DAPT treatment had no known association with processes related to HC fate repression or pro-sensory development (e.g. *Slc22a3, Slitrk6)* (Fig. 1b, blue). To confirm the microarray data, the differential expression of select genes was independently analyzed using RT-qPCR. For the top ranked DAPT down-regulated genes (FC ≤ −6σ; p value ≤ 0.05), the validation rate was more than 91% (22 out of 24 tested) (Table 1). To uncover the biological processes associated with these newly uncovered Notch-regulated genes, we performed gene ontology (GO) enrichment analysis using DAVID^24^,^25^. As expected, genes involved in mechanoreceptor differentiation and cell fate commitment were significantly enriched in the list of DAPT down-regulated genes (FC ≤ −1.215, p-value ≤ 0.07). GO enrichment analysis also revealed a previously unappreciated association of Notch signaling with cell-cell signaling, neurotransmitter-transport, synaptic transmission and signal transduction (Supplementary Table 2).

**Figure 2:**
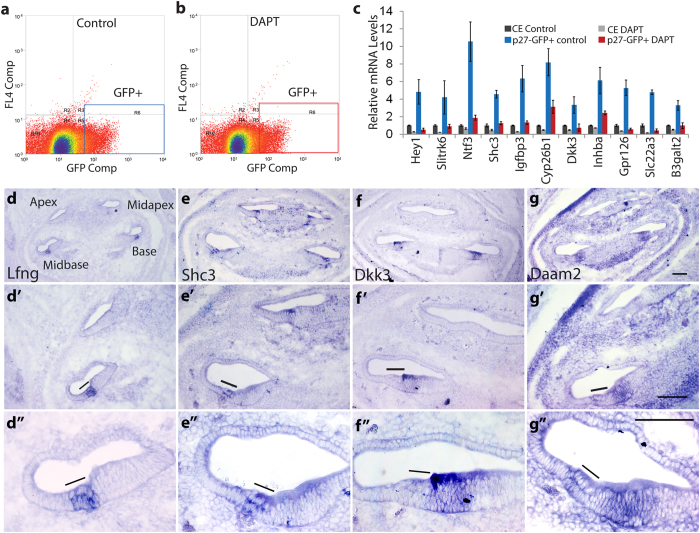
Identification of Notch-regulated genes that are selectively expressed in SCs **(a**–**c**) Analysis of Notch-dependent gene expression in purified SCs. **(a,b**) FACS plots of compensated GFP fluorescence of *p27*/*GFP* transgenic cochlear epithelial cells (CE) control (**a**) and CE DAPT (**b**) cells. Blue and red box indicate gating for GFP^+^ SCs. **(c)** RT-qPCR was used to analyze relative expression of Notch-regulated genes in CE control (black bar), CE DAPT (light grey bar), P27-GFP^+^ control (blue bar) and p27-GFP^+^ DAPT (red bar) SCs. Data expressed as mean ± SEM (n = 3, technical replicate). **(d**–**g”**) Cochlear expression pattern of newly identified Notch-regulated genes. Low, medium (‘) and high power images (“) of stage E15.5 (**d,d’,d”**) and E16.5 (**e**–**g”**) cochlear sections labeled for *Lfng* (**d,d’,d”**) *Shc3* (**e,e’,e”**), *Dkk3* (**f,f’,f”**) and *Daam2* (**g,g’,g”**) transcript. Black line marks sensory domain. Scale bar 100 μm.

In the *p27/GFP* reporter line GFP is specifically expressed in post-mitotic pro-sensory cells and differentiating SCs, which allows their purification by fluorescent activated cell sorting (FACS)^3^,^26^. To confirm Notch-dependent gene regulation selectively in differentiating SCs, we FACS-purified SCs (*p27/GFP*^+^) from control (Fig. 2a) and DAPT (Fig. 2b) treated *p27/GFP* transgenic cochlear explants stage E15.5 and analyzed gene expression using RT-qPCR. We selected *Slitrk6, Ntf3, Igfbp3, Cyp26b1*, *Inhba, Dkk3, B3galt2, Shc3, Gpr126 and Slc22a3* to be further analyzed. *Hey1*, a pro-sensory and SC-specific Notch target gene, functioned as a positive control. All genes tested were 2–4 fold higher expressed in FACS-purified SCs (*p27/GFP*^+^ control) than unfractionated cochlear epithelial cells (CE control) (Fig. 2c). Moreover, similar to the known Notch target gene *Hey1*, expression of *Slitrk6, Ntf3, Igfbp3, Cyp26b1, Dkk3, Inhba, B3galt2, Shc3, Gpr126* and *Slc22a3* was significantly reduced in SCs purified from DAPT-treated cochlear explants (*p27/GFP*^+^ DAPT) compared to SCs purified from control cochlear explants (*p27*/*GFP*^+^ control) (Fig. 2c), suggesting that Notch signaling positively regulates their SC-specific expression.

We next performed *in situ* hybridization (ISH) experiments on cochlear tissue stages E15.5–E16.5 to characterize the expression pattern of the newly identified Notch-regulated genes. At stage E15.5 and E16.5, HCs and SCs have already formed in the basal cochlear segment (base, mid-base); while in the more apical segment of the cochlea (mid-apex, apex) pro-sensory cells have yet to differentiate. Based on our ISH data as well as published expression data the majority of newly identified Notch-regulated genes can be grouped into two categories. The first category contains genes that are already highly expressed in undifferentiated HC and SC precursors as well as differentiating SCs as shown here for *Shc3* (Fig. 2e,e’) and as previously reported for *Slitrk6*^27^, *Ntf3*^28^ and *Cyp26b1*^29^. The second category contains genes that are upregulated during differentiation and are limited to differentiating SCs and/or cells of the greater epithelial ridge (GER) as shown here for *Lnfg* (Fig. 2d,d’), *Dkk3* (Fig. 2f,f’) and *Daam2* (Fig. 2g,g’) and as previously reported for *Igfbp3*^30^ and *Inhba*^31^.

### Canonical Notch signaling positively regulates SC-specific genes *in vivo*

**Figure 3:**
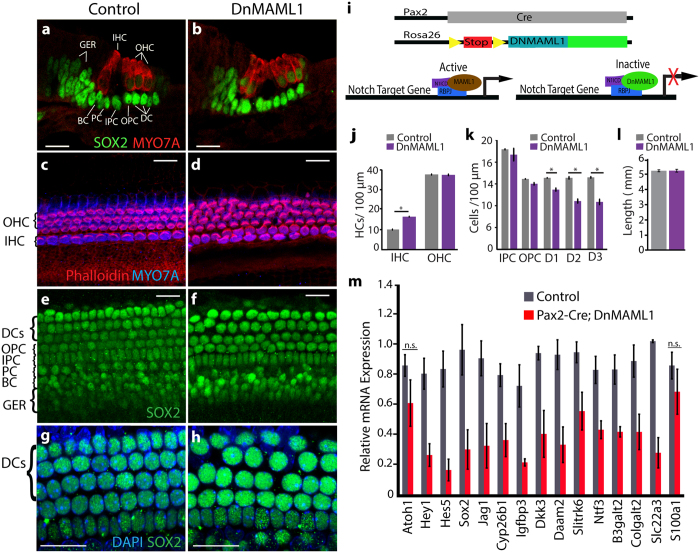
Newly identified Notch-regulated genes are significantly reduced in *DnMAML1* cochleae. (**a–h**) HC and SC phenotype of *Pax2-Cre; ROSA26*^*DnMAML1*/+^ (DnMAML1) transgenic and wild type (control) littermates stage E18.5 was analyzed in the cochlear base. Abbreviations: inner HCs (IHC), outer HCs (OHC), border cell (BC), phalangeal cell (PC), inner pillar cells (IPC), outer pillar cells (OPC), and Deiters’ cells (DC), greater epithelial cells (GER). Scale bars for all panels, 20 μm. (**a,b**) Representative confocal images of cross-sections through control (**a**) and DnMAML1 expressing (**b**) cochleae; Anti-myosinVIIa (MYO7A, red) labels HCs, anti-SOX2 (SOX2, green) labels SCs and GER cells. **(c**–**f)** Maximum z-projections of control (**c,e**) and DnMAML1 expressing (**d,f**) HC (**c,d**) and corresponding SC layer (**e,f**). MYO7A (blue) labels HCs; phalloidin (red) labels actin-rich HC bundles, SOX2 (green) labels SC and GER cell nuclei. **(g,h)** Maximum z-projections of control (**g**) and DnMAML1 expressing (**h**) outer SC layer. SOX2 (green) labels Deiters’ cell and pillar cell nuclei, DAPI staining (blue) labels cell nuclei. **(i)** Schematics showing how forced DnMAML1 expression attenuates canonical Notch signaling. **(j**–**l)** Quantification of HC (**j**) and SC (**k**) density and cochlear length (**l**) of the control and DnMAML1 expressing cochleae stage E18.5. Inner (IHC) and outer HC (OHC) density as well as outer SC (IPC, OPC, D1, D2, D3) density was analyzed in the cochlear base. Data expressed as mean ± SEM (n = 3, *p ≤ 0.05 was considered significant). **(m)** Notch target gene expression in DnMAML1 expressing (red bar) and control cochlear epithelia (grey bar) stage E18.5. Relative transcript levels were analyzed using RT-qPCR. Data expressed as mean ± SEM (n = 3 biological replicates; p-value > 0.05 was considered not significant (n.s.)).

Although GSIs like DAPT are widely used to inhibit Notch signaling, some of the observed changes in gene expression may be due to the inhibition of GS-dependent processes other than Notch signaling^32^. To independently confirm that canonical Notch signaling is indeed required for the regulation of the newly identified DAPT down-regulated genes, we chose to use the dominant-negative Master Mind-Like1 (DnMAML1) mouse line to specifically inhibit Notch mediated transcriptional activation in the developing cochlea. Upon Cre mediated excision of a stop cassette, the *ROSA26* promoter drives the expression of a truncated form of the human MAML1 protein fused to GFP^33^. This fusion protein forms a transcriptionally inactive complex with NICD and RBP-J, and competition with the wild-type MAML proteins abrogates Notch signaling elicited from all possible Notch ligand-receptor interactions (Fig. 3i). We used the *Pax2-Cre* line to drive inner ear-specific expression of DnMAML1^34^. *Pax2-Cre; ROSA26*^*DnMAML*1/+^ animals were examined at E18.5 to circumvent neonatal lethality. At E18.5, HC and SC differentiation is largely completed, and in wild type (control) cochleae myosinVIIa (MYO7A) expressing HCs are arranged in three rows of outer HCs and one row of inner HCs (Fig. 3a,c). The length of DnMAML1 expressing cochleae was unchanged compared to control (Fig. 3l); however, HCs were miss-patterned (Fig. 3b,d) and the number of inner HCs was significantly increased compared to control (Fig. 3j,l). Moreover, in contrast to the uniform orientation of actin-rich HC bundles seen in control cochleae, HC bundles were severely disoriented in DnMAML1 expressing cochleae (Fig. 3c,d). At E18.5 SCs in the base are largely differentiated and based on the morphology and location of their SOX2^+^ nuclei can classified as border cells, inner phalangeal cells, inner and outer pillar cell and Deiters’ cells (Fig. 3a,e). SCs were largely retained in DnMAML1 expressing cochleae (Fig. 3b,f); however Deiters’ cell nuclei were enlarged and the density of basally located Deiters’ cells was modestly reduced in DnMAML1 expressing cochleae compared to control (Fig. 3g,h,k).

To determine whether the newly identified DAPT down-regulated genes are positively regulated by Notch signaling *in vivo*, we isolated cochlear epithelia from E18.0 *Pax2-Cre; ROSA26*^*DnMAML1*/+^ embryos and wild type (control) littermates, prepared RNA and performed RT-qPCR to analyze gene expression. The known Notch target genes *Hes5, Hey1, Sox2* and *Jag1* served as positive controls. As expected, we found that *Hes5, Hey1, Sox2* and *Jag1* transcripts were significantly down-regulated in the DnMAML1 expressing cochlear epithelia, whereas the expression of the SC marker gene *S100a1* was not significantly reduced^35^ (Fig. 3m). Furthermore, our RT-qPCR experiments revealed that all the examined genes (*Slitrk6, Ntf3, Igfbp3, Cyp26b1, Dkk3, Daam2, Shc3, B3galt2, Colgalt2* and *Slc22a3)*, were significant down-regulated in DnMAML1 expressing cochlear epithelia as compared to wild type cochlear epithelia (Fig. 3m), indicating that canonical Notch signaling is required to maintain these SC-specific genes *in vivo*.

### Ectopic Notch signaling activates a SC-specific gene expression program

Previous studies revealed that over-expression of the Notch1 intracellular domain (N1ICD) is sufficient to induce ectopic sensory patches that contain both HC and SC-like cells in non-sensory otic regions at early stages of otic development^22^,^36^,^37^. However, after E12.5 activation of Notch signaling in non-sensory cochlear epithelial cells is not sufficient to induce ectopic HCs, even though the pro-sensory/SC-specific gene *Sox2* is induced in N1ICD expressing cell^38^,^39^,^40^. We examined whether Notch over-activation after E12.5 is sufficient to induce the newly identified Notch-regulated genes. Specifically, we used the *Emx2*^*Cre*/+^ line^41^, which turns on *Cre* expression at around E13.5^16^, and allows for ectopic expression of *N1ICD (ROSA26*^*N1ICD*^)^42^ throughout the cochlear epithelial duct.

**Figure 4:**
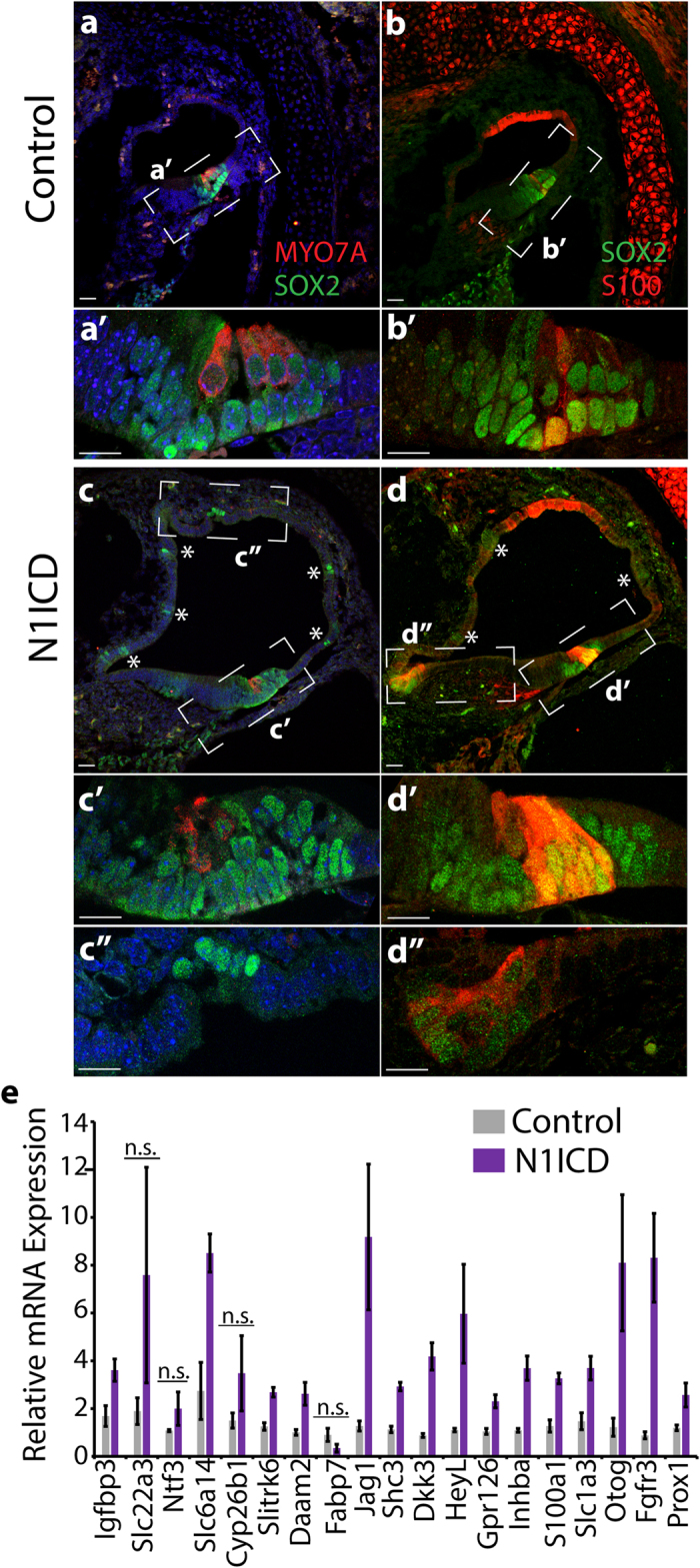
N1ICD overexpression induces SC-specific gene expression. (**a–d”**) HC (**a,c**) and SC phenotype (**b,d**) of *Emx2*^*Cre*/+^*; ROSA26*^*N1ICD*/+^ mutants (N1ICD; (**c,d**) and their wild type (control; (**a,b**) littermates stage E18.0 was analyzed in adjacent cochlear sections. HCs are marked by anti-myosinVIIa (MYO7A, red) staining, Anti-SOX2 staining (green) marks SCs and GER cells within the sensory domain. Deiters’ cells and pillar cells are marked by anti-SOX2 (SOX2, green) and anti-S100 (S100, red) staining. Hoechst staining (blue) labels cell nuclei. Dashed white lines indicate location of corresponding high power confocal images (‘,”). No ectopic HCs are observed in N1ICD over-expressing cochlea (**c”**); however the number of outer HCs is reduced (**c,c’**) compared to control (**a,a’**) and ectopic S100^+^ SOX2^+^ cells are observed in the outer HC domain (**d’**) and non-sensory epithelium (**d”**) but not in control (**b,b’**). **(e)** RT-qPCR analysis of SC-specific gene expression in cochlear epithelia obtained from E18.0 *Emx2*^*Cre*/+^*; ROSA26*^*N1ICD*/+^ mutants (N1ICD, purple bars) and wild-type littermates (control, grey bars). Data expressed as mean ± SEM (n = 3, biological replicates, p-value > 0.05 was considered not significant (n.s.)). Similar results were obtained in a second independent experiment.

As *Emx2*^*Cre*/+^*; ROSA26*^*N1ICD*/+^ (N1ICD) animals die at birth, we limited our analysis to late embryonic stages (E18.0–E18.5). The cochlear lumen of N1ICD over-expressing cochleae was severely enlarged and malformed compared to wildtype control cochleae (Fig. 4a–d). As expected, we found scattered clusters of ectopic SOX2^+^ cells throughout non-sensory regions of N1ICD overexpressing cochlear duct. Consistent with previous reports, the ectopic SOX2^+^ cell clusters were void of ectopic MYO7A^+^ HCs (Fig. 4c,c”). Interestingly, in contrast to the normal compliment of three outer HCs seen in wild type (control) cochleae (Fig. 4a,a’), outer HCs were frequently missing in the N1ICD over-expressing cochleae, ranging from only two outer HCs to no outer HCs (Fig. 4c,c’). To determine whether Notch activation is sufficient to drive the expression of the newly identified Notch-regulated genes, we isolated cochlear epithelia from E18.0 N1ICD mutant embryos and wild type (control) littermates, prepared RNA and used RT-qPCR to analyze gene expression in these RNA samples. Notch target genes *HeyL* and *Jag1* functioned as positive controls. 11 out of the 12 newly identified Notch-regulated genes tested showed an increase in gene expression as a result of ectopic activation of Notch signaling. The expression of *Igfbp3, Slc6a14, Slitrk6, Daam2, Shc3, Dkk3, Gpr126* and *Inhba* was significantly increased in response to Notch over-activation; *Scl22a3, Ntf3* and *Cyp26b1* expression was increased, but the level of induction varied substantially across the examined N1ICD samples (Fig. 4e). Next we analyzed the expression of genes that are characteristic of SCs (*S100a1*^35^*, Slc1a3* (GLAST)^43^, *Otog*^44^*, Prox1*^45^ and *Fgfr3*^46^). The expression of these SC-specific marker genes was significantly up-regulated in N1ICD over-expressing cochlear epithelia compared to control cochlear epithelia (Fig. 4e). These findings suggest that Notch signaling is sufficient to activate a SC-specific gene expression program in the differentiating cochlea.

To determine whether Notch over-activation produced ectopic SC-like cells we stained E18.0 wild type and N1ICD over-expressing cochlear tissue with a pan S100 antibody, which in the neonatal cochlea marks Deiters’ cells and pillar cells^47^. In control cochlear tissue, anti-S100 staining marked SOX2^+^ pillar cells and SOX2^+^ Deiters’ cells; outside the sensory epithelium anti-S100 staining marked SOX2^−^ cells of the presumptive stria vascularis (Fig. 4b,b’)^48^. In the N1ICD over-expressing cochleae, ectopic anti-S100 staining was observed in the HC layer atop of pillar cells and Deiters’ cells (Fig. 4d,d’). Furthermore, in N1ICD over-expressing cochlear epithelia, due to the increase in outer SCs and the decrease in outer HCs, the outer SC/outer HC ratio was significantly increased compared to wild type cochlear epithelia (N1ICD: 8.10 ± 2.42; control:1.71 ± 0.02, n = 3, p-value ≤ 0.05) (Supplementary Table S3). It is possible that outer HCs death contributes to the observed N1ICD mutant phenotype. For instance, adenoviral overexpression of PROX1, a known Notch-regulated SC-specific transcription factor causes the death of outer HCs through inhibiting the expression of HC-specific transcription factor GFI1^49^. However, recent studies found no defects in outer HC survival when Notch signaling was ectopically activated in differentiating HCs^50^,^51^. These findings and our observation that the decrease in the number of outer HCs is accompanied by an increase in the number of outer SCs, suggests that outer HC precursors switched fate and differentiated into outer SC-like cells. We also observed infrequently clusters of SOX2^+^ S100^+^ cells outside the sensory epithelium, however the majority of ectopic SOX2^+^ clusters were S100^−^ (Fig. 4d,d”). Taken together, these data suggest that Notch signaling is sufficient to ectopically induce a SC-like fate in undifferentiated HC precursors and in a subset of cochlear epithelial cells.

### Reduction in canonical Notch signaling results in progressive Deiters’ cell loss

**Figure 5:**
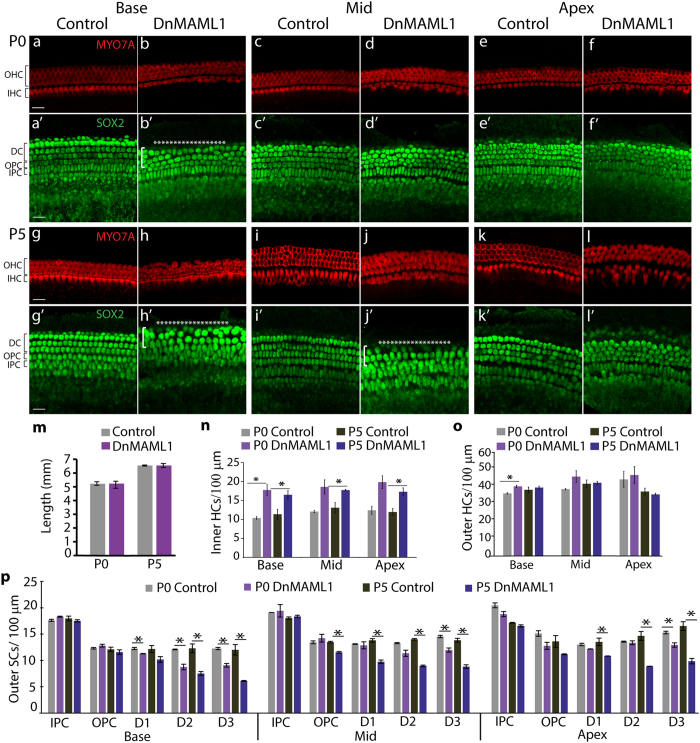
Expression of DnMAML1 results in a progressive loss of Deiters’ cells. HC and SC phenotype in *Emx2*^*Cre*/+^ *ROSA26*^*DnMAML1*/+^ (*DnMAML1*) transgenics and their wild type (control) littermates, stage P0 and P5. **(a-l’)** Maximum z-projections of HC layer (MYO7a, red) and corresponding SC layer (‘) (SOX2, green) in control (P0: (**a,c,e**); P5: (**g,i,k**) and *DnMAML1* (P0: (**b,d,f**); P5: (**h,j,l**) cochleae. Shown are basal (**a-b’,g-h’**), mid (**c-d’,i-j’**) and apical (**e-f’,k-l’**) fields. White asterisks in B’, H’ and J’ indicate missing Deiters’ cells. **(m)** Quantification of total length of control and DnMAML1 expressing cochleae stage P0 and P5. **(n**–**p)** Quantification of inner HC (**n**), outer HC (**o**) and outer SC (**p**) density in the base, mid and apex of control (P0, light grey; P5 dark grey) and DnMAML1 expressing (P0 light purple; P5 dark purple) cochleae. Abbreviations: inner pillar cells (IPC), outer pillar cells (OPC), and Deiters’ cells row 1, 2 and 3 (DC1-3). Data expressed as mean ± SEM (n = 3, biological replicates, *p ≤ 0.05 was considered significant). Scale bars for all panels, 20 μm.

To determine whether physiological levels of Notch signaling are required for proper SC development, we re-examined the SC phenotype in DnMAML1 expressing cochleae. Our initial analysis of stage E18.5 *Pax2-Cre; ROSA26*^*DnMAML1*/+^ animals revealed mild defects in SC patterning and morphology and a mild reduction in the number of basally located outer SCs, namely Deiters’ cells. One possibility for the loss of Deiters’ cells is SC-to-HC conversion; however, the reduction in Deiters’ cells in the DnMAML1 expressing cochleae was not accompanied by an increase in outer HCs (Fig. 3j,k). To determine whether defects in Deiters’ cell development become more pronounced at later stages we modified our experimental approach. We examined *Emx2*^*Cre*/+^*; Rosa26*^*DnMAML1*/+^ animals, which in contrast to *Pax2-Cre; ROSA26*^*DnMAML1*/+^ animals, survive past birth, allowing us to characterize SCs as they undergo postnatal differentiation and maturation. As observed in the *Pax2-Cre; ROSA26*^*DnMAML1*/+^ late embryonic cochleae, cochlear length was unchanged in *Emx2*^*Cre*/+^*; ROSA26*^*DnMAML1*/+/+^ postnatal cochleae (P0 and P5) compared to wild type (control) littermates (Fig. 5m). However, as already observed in *Pax2-Cre; ROSA26*^*DnMAML1*/+^ cochleae, HCs were miss-patterned in the *Emx2*^*Cre*/+^*; ROSA26*^*DnMAML1*/+^ postnatal cochleae (P0 and P5), and the number of inner HCs was significantly increased compared to wild type (control) littermates, whereas the number of outer HCs remained relative unchanged (Fig. 5a–l,n,o). In contrast to the relative stable HC phenotype, the outer SC phenotype significantly worsens between P0 and P5 in DnMAML1 expressing cochleae (Fig. 5a’–l’). At P0, Deiters’ cell loss was only evident in the base of the DnMAML1 expressing cochleae (Fig. 5b’). However, five days later at P5, Deiters’ cells were missing throughout the length of the cochlear duct in DnMAML1 expressing cochleae and the number of 2^nd^ and 3^rd^ row Deiters’ cells was significantly reduced compared to P5 control cochleae (Fig. 5p). Deiters’ cells in P5 DnMAML1 expressing cochleae had enlarged cell nuclei and their nuclear arrangement was disorganized (Fig. 5h’), a large contrast to the stereotypical arrangement of wild-type Deiters’ cell nuclei (Fig. 5g’), suggesting that prolonged reduction in canonical Notch signaling results in progressive degeneration of Deiters’ cells. What could be the molecular basis for the observed defects? In DnMAML1 expressing cochleae *Jag1* expression is severely reduced (Fig. 3m). It has been previously shown that early otic deletion of *Jag1* or a reduction in JAG1 activity causes complex auditory defects; inner HCs are overproduced, whereas outer HCs and their accompanying Deiters’ cells are reduced in number or largely missing^8^,^9^,^52^,^53^,^54^. To examine the function of JAG1 in the differentiating cochlea, we conditionally deleted *Jag1* using the *Emx2*^*Cre*/+^ line and a previously developed *Jag1* floxed line^8^. Ablation of *Jag1* at ~E13.5 resulted in similar auditory defects as previously reported for early otic ablation paradigms. The *Jag1* mutant (*Emx2*^*Cre*/+^; *Jag1*^*^Δ/Δ^*^) sensory epithelia contained doublets of inner HCs throughout the length of the cochlear duct and contained few scattered outer HCs in the midbase. Moreover, Deiters’ cells were largely missing from the *Jag1* mutant cochleae (Supplementary Fig. S1). These findings suggest that JAG1 function is critical for the differentiation of the lateral pro-sensory domain. Thus, the severely reduced expression of *Jag1* in DnMAML1 expressing cochleae might be the basis for the abnormal Deiters’ cells differentiation and eventual Deiters’ cell loss.

### Ablation of canonical Notch signaling causes differentiating Deiters’ cells to die

**Figure 6:**
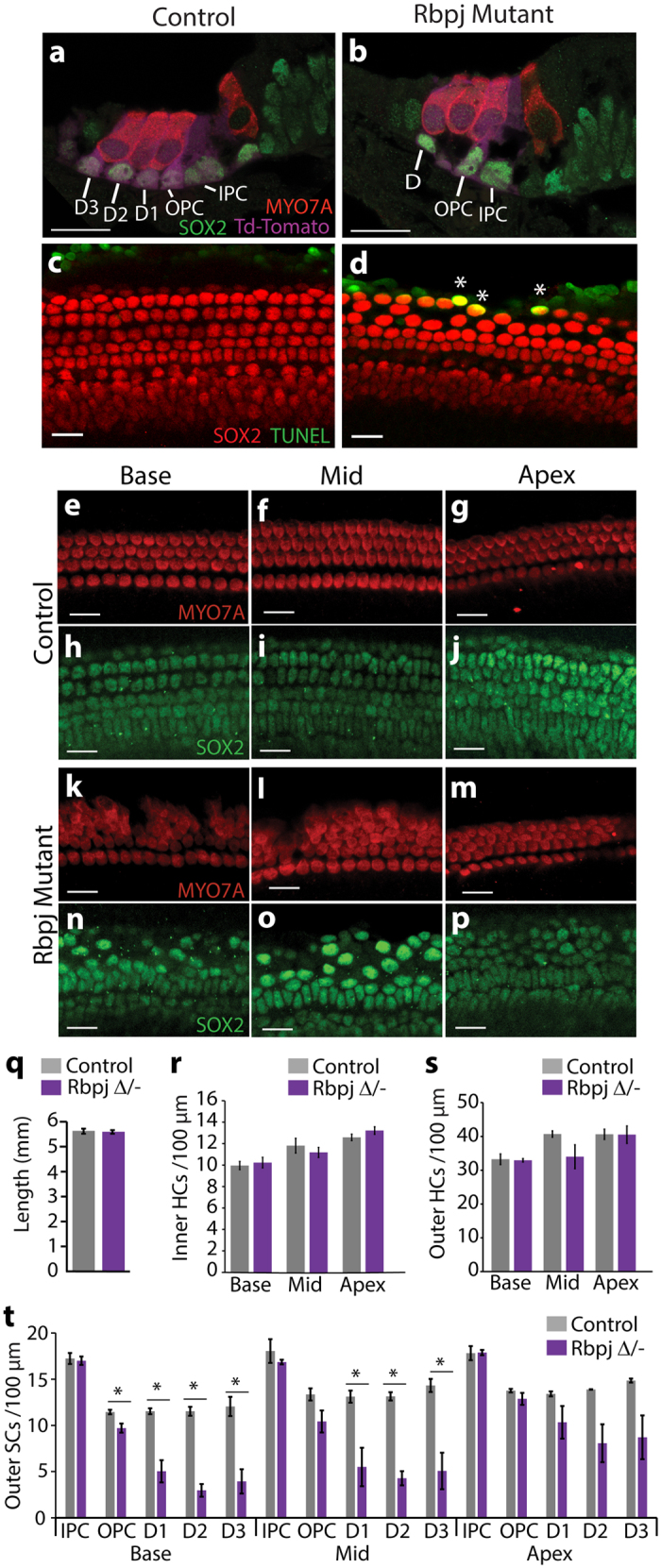
Ablation of *Rbpj* results in Deiter’s cell death. Tamoxifen was injected at E14.5 and E15.5 and cochlear HC (MYO7A) and SC phenotype (SOX2) in *Fgfr3-icreER; Ai14; Rbpj*^Δ/+^ (control) and of *Fgfr3-icreER; Ai14; Rbpj*^Δ/−^ (Rbpj mutants) littermates was analyzed at stage E18.5. **(a,b)** Representative confocal images of mid-basal cochlear sections from control (**a**) and *Rbpj* mutant (**b**) animals. Ai14 Cre-reporter (Td-Tomato, purple) reveals cells in which Cre-mediated recombination occurred. Note that Deiters’ cells (D1-D3) but not outer pillar cells (OPC) or inner pillar cells (IPC) are missing in *Rbpj* mutants (**B**). **(c,d)** Representative confocal images of SC layer in control (**c**) and *Rbpj* mutant (**d**) cochlear surface preparations. Note mid-basal segments are shown. SOX2 marks SC nuclei (red) TUNEL staining reveals apoptotic/necrotic cell nuclei (green). Asterisks mark TUNEL^+^ Deiters’ cells in the *Rbpj* mutant (**d**) cochlear sensory epithelia. (**e**–**p**) Maximum z-projections of HC layer (MYO7A, red) and SC layer (SOX2, green) of control (**e**–**j**) and *Rbpj* mutant (**k**–**p**) cochlear surface preparations at the indicated position. **(q**–**t**) Quantification of total cochlear length (**q**) and inner HC (**r**), outer HC (**s**) and outer SC (IPC, OPC, D1-3) (**t**) density in the cochlear base, mid and apex of control and *Rbpj* mutant animals. Data expressed as mean ± SEM (n = 3, biological replicates, *p ≤ 0.05 was considered significant). Scale bars for all panels, 20μm.

To independently confirm the requirement of canonical Notch signaling for proper Deiters’ cell development, we decided to abolish Notch-mediated transcriptional activation selectively in differentiating outer SCs. The transcription factor RBP-J is critical for the transcriptional output of Notch (1-4) receptor signaling and ablation of the *Rbpj* gene abolishes canonical Notch signaling within that cell. Early otic deletion of *Rbpj* results in severe defects in auditory sensory development due to defects in pro-sensory maintenance and or HC precursor survival respectively^38^,^55^. To bypass the early requirement for RBP-J we selectively deleted the *Rbpj* gene at later stages in differentiating Deiters’ cells and pillar cells using the recently developed tamoxifen inducible *Fgfr3-iCreER* transgenic line^56^. In the differentiating cochlea *Fgfr3-iCreER* is similar to endogenous *Fgfr3* highly expressed in the lateral sensory domain, including pillar cells, Deiters’ cells and outer HCs^57^. Tamoxifen was administrated at E14.5 and E15.5 to pregnant dams, and the HC and SC phenotype was analyzed three days later at stage E18.5 in control (*Fgfr3-iCreER; Rbpj*^Δ/+^) (Fig. 6a,c,e–j) and *Rbpj* mutant *(Fgfr3-iCreER; Rbpj*^Δ/−^) littermates (Fig. 6b,d,k–p). Deletion of *Rbpj* did not alter cochlear length (Fig. 6q). However, SOX2 staining revealed large gaps/holes in the *Rbpj* mutant SC layer, which corresponded to missing Deiters’ cells (Fig. 6b,d,n,o). The observed loss in Deiters’ cells in *Rbpj* mutant cochleae was not accompanied by an increase in outer or inner HCs (Fig. 6r–t). However, due to the loss of surrounding Deiters’ cells, outer HCs clumped together, and outer HC arrangement was severely disorganized in the base and mid segment of the *Rbpj* mutant cochleae (Fig. 6k,l). Moreover, TUNEL staining revealed apoptotic cells within the outer SC layer in *Rbpj* mutant, but not in control cochlear tissue, suggesting that in the absence of *Rbpj*, Deiters’ cell survival is compromised (Fig. 6c,d). The remaining *Rbpj* mutant Deiters’ cells had severely enlarged cell nuclei, indicating cellular stress and/or injury (Fig. 6d,n,o). Interestingly, pillar cells, particularly inner pillar cells, were largely unaffected by the loss of *Rbpj* (Fig. 6b,s). FGFR3 signaling, which is highly activated in differentiating pillar cells plays a key role in their differentiation^58^,^59^, and it is likely that FGFR3 signaling largely compensated for the loss of Notch signaling in pillar cells.

## Discussion

Despite the importance of canonical Notch signaling for vertebrate development, only a limited number of Notch target genes have been identified and characterized in various tissues. In the developing inner ear, genes functioning in pro-sensory cell development (*Sox2, Jag1, and Fgf20*) and HC fate repression (*Hey1, Hey2, HeyL, Hes1, Hes5*) have been shown to be transcriptionally regulated/co-regulated by Notch signaling^60^. Our study identifies a new class of Notch regulated SC-specific genes, with functions largely unrelated to pro-sensory development and HC fate repression. Among these genes are known direct Notch target genes *Gucy1a3, Gucy1b3, Inhba*^61^*, Fabp7*^62^, *Igfbp3*^63^*, and Nrarp*^64^. However, for the majority of genes it is unknown whether Notch signaling acts directly or indirectly on their transcription and future studies are warranted. Among the newly identified Notch-regulated genes are genes that play key roles in cell signaling pathways including Wnt (*Dkk3*^65^*, Daam2*^66^), Igf1r (*Igfbp3*^67^), Activin (*Inhba*^68^) and retinoic acid (*Cyp26b1*^69^) signaling, revealing a previously unappreciated level of cross-talk between Notch signaling and these developmentally important signaling pathways. Also among the top ranked Notch-regulated genes are genes that are critical for cochlear innervation (*Ntf3* and *Slitrk6*)^27^,^70^ as well as synaptogenesis (*Ntf3*)^71^, implicating a regulatory role for Notch signaling in these developmental processes. In addition, the presence of genes that function in amino acid/neurotransmitter transport (*Slc6a14*^72^*, Slc22a3*^73^) and nitric oxide/cGMP signaling (*Gucy1a3, Gucy1b3*)^74^, suggests that Notch signaling might function in SC physiology and cochlear homeostasis.

Is Notch signaling sufficient to induce a SC fate? Our analysis of the *Emx2*^*Cre*/+^*; ROSA26*^*N1ICD*/+^ mutant cochleae suggests that Notch signaling promotes a SC-specific gene expression program, but the ability of Notch signaling to ectopically induce SC-like-cells is highly cell context dependent. Previous studies showed that Notch over-activation in undifferentiated inner ear sensory epithelia significantly reduces the number of HCs^22^,^37^,^75^. Here, we provide evidence that ectopic Notch activation in outer HC precursors not only represses a HC-specific program, but instructs outer HC precursors to differentiate into S100^+^ SOX2^+^ outer SC-like cells. In contrast to outer HCs, inner HCs form relatively normal in *Emx2*^*Cre*/+^*; ROSA26*^*N1ICD*/+^ mutant cochlea. Inner HCs are the first cells to differentiate, and it is likely that these cells have already initiated a HC-specific program at the time of Notch over-activation, rendering them unresponsive to Notch-mediated reprogramming. Support for this idea comes from two recent studies, which show that ectopic Notch activation in differentiating auditory HCs is not sufficient to halt the initial phase of their HC-specific program^39^,^51^. Outside the sensory epithelium we only infrequently observed ectopic patches of SC-like cells, suggesting the existence of inhibitory factors/signals that might actively repress the conversion of these cells into SC-like cells.

Is canonical Notch signaling required for SC development? Our analysis revealed that prolonged reduction (*DnMAML1* transgenic) or ablation of canonical Notch signaling (*Rbpj* mutant) resulted in a selective loss of outer SCs (Deiters’ cells). The progressive loss of Deiters’ cells was not due to SC-to-HC conversion as no corresponding increase in the number of outer or inner HCs was observed. Instead, the gradual decrease in the number of Deiters’ cells in DnMAML1 expressing cochleae, as well as the presence of TUNEL positive Deiters’ cells in *Rbpj* mutants suggest that attenuation or loss of canonical Notch signaling causes differentiating Deiters’ cells to initiate an apoptotic or necrotic-like process and die. Why do Deiters’ cells die in the absence of canonical Notch signaling? One plausible explanation is that in the absence of Notch signaling genes, critical for SC differentiation, are absent, causing cell stress and subsequent cell death. Alternatively, Notch signaling may be required for the expression of a pro-survival gene(s), which once lost results in cell death. Interestingly, in *Rbpj* mutant and in DnMAML1 expressing cochleae basal Deiters’ cells were more affected than Deiters’ cells located further apically. Furthermore, Deiters’ cells located at the lateral edge of the sensory epithelium were more affected than more medially located Deiters’ cells. This graded response suggests the existence of additional signals that modulate Notch-dependency in differentiating SCs. Candidates are FGFR signaling and Sonic Hedgehog signaling, which have been recently reported to modulate Notch-dependent gene expression in pillar cells and apical pro-sensory cells respectively^13^,^76^.

The observed Deiters’ cell survival defect and the lack of ectopic outer HCs in *DnMAML1* transgenics and *Rbpj* mutants is in stark contrast to the HC and SC phenotypes in *Notch1 mutants and Dll1/Jag2* compound mutants; in these mutants outer HCs are overproduced at the cost of Deiters’ cells^11^. How can these differences be explained? It could be reasoned that the observed defects in *Rbpj* mutants and *DnMAML1* transgenics are due to the disruption of functions independent of the Notch signaling pathway. In the absence of NICD, RBP-J functions as a repressor, thus loss of *Rbpj* not only disrupts Notch-mediated gene activation but leads to an upregulation of genes normally repressed by RBP-J. In addition, both RBP-J and the MAML co-factors are known to participate in transcriptional activator complexes independent of NICD^77^,^78^. However, to date, no overlap in the Notch-independent functions of RBP-J and MAML proteins has been reported, making it extremely unlikely that the defects in SC survival observed in *Rbpj* mutants and *DnMAML1* transgenics were caused by the disruption of Notch-independent processes.

There are some similarities between the cochlear phenotypes of *Rbpj* mutants, *DnMAML1* transgenics and *Jag1* mutants. In contrast to early otic ablation of *Notch1*, early otic ablation of the *Jag1* results in ~2-fold increase in the number of inner HCs, but a severe reduction in the number of outer HCs and their surrounding Deiters’ cells^8^,^9^,^52^. The reduced number of outer HCs and Deiters’ cells observed in *Jag1* mutants has been attributed to defects in pro-sensory domain formation^9^; however this interpretation has been called into question by a more recent study that found no defects in pro-sensory domain formation or maintenance after early otic-specific deletion of *Jag1*^38^. We found that ablation of *Jag1* just prior to cochlear HC differentiation results in a similar cochlear phenotype than early otic deletion, suggesting that JAG1-activated Notch signaling is required for proper differentiation of the lateral sensory domain rather than its specification or maintenance. Thus, the attenuation/loss of JAG1 activated Notch signal in DnMAML1 expressing and *Rbpj* mutant cochleae might be a contributing factor for the seen defects in Deiters’ cell development.

Why is the HCs and SC phenotype observed in *DnMAML1* transgenics and *Rbpj* mutants different from *Notch1* mutants? Recent studies revealed that differentiating SCs express two additional Notch receptors, namely *Notch2* and *Notch3*^79^,^80^; their cochlear functions have not yet been described. It is possible that the co-expressed Notch receptors, activated by distinct sets of Notch ligands, mediate unique functions in differentiating SCs with DLL1/JAG2-NOTCH1 mediating the HC-repressive function of Notch signaling and JAG1-NOTCH2/3 mediating the instructive function(s) of Notch signaling. There is some precedence for selective roles of co-expressed Notch receptors. For instance, in vascular smooth muscle cells co-expressed NOTCH2 and NOTCH3 have unique functions in regulating cell proliferation and survival^81^.

Alternatively, it is possibly that a different signal threshold is required for the instructive and inhibitory functions of Notch signaling and that in the absence of NOTCH1 - the dominant receptor with the presumed highest signal strength - NOTCH2/3 signaling is sufficient to sustain Deiters’ cell differentiation/survival but is insufficient to sustain HC-fate repression. This model is compatible with previous findings that showed that low concentrations of GSI DAPT (1–5 μM) are sufficient to produce a *Notch1* mutant-like phenotype, whereas high concentrations of DAPT (30–100 μM) are required to produce a *Rbpj* mutant-like phenotype in embryonic cochlear explants^82^,^83^.

## Materials and Methods

### Mouse breeding and genotyping

The Animal Care and Use Committees of Johns Hopkins University approved all of the protocols performed in this study and the methods were carried out in accordance with the approved guidelines. The *P27-GFP* BAC transgenic line was obtained from Neil Segil (USC, USA)^3^. The *Pax2-Cre* BAC transgenic line was obtained from Andrew Groves (Baylor College, USA)^34^. The *Emx2*^*Cre*/+^ knock in line was obtained from Shin Aizawa (RIKEN, Japan)^41^. The Cre inducible *ROSA26*^*Dn-MAML1*/+^ line was obtained from Warren Pear (University of Pennsylvania, USA). The Cre inducible *ROSA26*^*N1ICD*/+^ line^42^ (#008159) and Ai14 TdTomato Cre reporter line^84^ (#007914) was purchased (Jackson Laboratory). The *Fgfr3-iCreER* PAC transgenic line was obtained from William Richardson (UCL, UK)^85^. Conditional *Rbpj*^*fx/fx*^ and conventional *Rbpj*^*−*/+^ knock out lines were obtained from Tasuku Honjo (University of Kyoto, Japan)^86^. Conditional *Jag1* line was obtained from Julian Lewis (Cancer Research UK, UK)^8^. Mice were genotyped by PCR as previously described for each line. Mice of both sexes were used in this study. All mouse lines were maintained on a mixed background of C57BL/6 and CD-1. To conditionally delete the floxed *Rbpj* allele, pregnant dams received a single injection of tamoxifen (0.125 mg/g body weight, Sigma) and progesterone (0.125 mg/g body weight, Sigma) at E14.5 and E15.5.

### Organotypic culture

E15.5 *P27/GFP* transgenic embryos were harvested in 1X HBSS (Corning Cellgro). After isolation, cochlear tissue was enzymatically treated (see below) to free the cochlear duct and its innervating spiral ganglion from surrounding tissue. The cochlear duct, attached mesenchyme, and innervating spiral ganglion was placed onto filter membranes (SPI Supplies, Structure Probe) and cultured in DMEM/F12 (Corning Cellgro) supplemented with 2.5 ng/ml EGF (Sigma), 2.2 ng/ml FGF (Sigma), 1X N2 supplement (Life Technologies), 100 U/ml Penicillin (Sigma). Cultures were maintained at 37 °C in a 5% CO_2_/20% O_2_ humidified incubator. At plating half of the cultures received DAPT (GSI) or DMSO (vehicle control). 25 mM stock solution of DAPT (*N*-[(3, 5-Difluorophenyl) acetyl]-L-alanyl-2-phenyl] glycine-1,1-dimethylethyl ester) (Tocris Bioscience) was applied at a final concentration of 3.33 μM. DMSO (Sigma) was applied at a final concentration of 0.013%. After culture, DMSO-treated cochlear explants and DAPT-treated cochlear explants were separately pooled (6–10 each) and cochlear epithelia isolated (see below).

### Cochlear epithelial preparations

Cochlear tissue was washed in CMF-PBS and incubated in dispase (1 mg/ml; Life Technologies) and collagenase (1 mg/ml; Worthington) for 8 minutes. After a 30 minute incubation in 10% FBS in DMEM-F12, non-epithelial tissue was removed by manual dissection with 30-gauge needles. For stages E17.5 and older, the cochlear capsule and the spiral ganglion were removed prior to dispase/collagenase treatment.

### Cell sorting

FACS-based purification of p27/GFP^+^ SCs was carried out on a MOFLO cytometer (DAKO-Cytomation), with a 100-μm CytoNozzle. Cell dissociation and FACS-based cell sorting was performed as previously described^3^.

### RNA isolation, microarray and RT-qPCR experiments

Total RNA was extracted from cochlear epithelial cells or purified SCs using the RNeasy Micro Kit (QIAGEN). For qPCR based validation experiments mRNA was reverse transcribed into cDNA using the iScript cDNA synthesis kit (Bio-Rad). SYBR Green based qPCR was performed using Fast SYBR® Green Master Mix reagent (Applied Biosystems) and gene-specific primers. Relative gene expression was analyzed using the CT method^87^. The ribosomal gene *Rpl19* was used as an endogenous reference gene and wild type early postnatal cochlear tissue was used as calibrator. Q-PCR primers used in this study are listed in Supplementary Table S4. Microarray experiments were performed on three biological replicate RNA samples per condition. Total RNA was labeled using Ambion® Expression WT kit (Life Technologies). Labeled RNA was hybridized onto GeneChip® Mouse Exon 1.0 ST Arrays (Affymetrix) and chips were scanned and analyzed according to manufactures manuals. GeneChip Expression Affymetrix CEL files were extracted and data normalized with the Partek GS 6.6 platform (Partek Inc.). Partek’s extended meta-probe set was used with RMA normalization to create quantile-normalized log2 transcript signal values, which were used in subsequent ANOVA analyses. The microarray data is deposited in the Gene Expression Omnibus (GEO) data base, accession number GSE67085.

### Tissue processing

Embryonic and early postnatal animals were staged using the EMAP eMouse Atlas Project (http://www.emouseatlas.org) Theiler staging criteria. For sectioning, whole heads (Stages E15-E17) or dissected inner ears (Stage E18 –P5) were fixed in 4% para-formaldehyde (PFA) in 1X PBS overnight, put through a sucrose gradient (10% sucrose for 30 minutes, 15% sucrose for 30 minutes and 30% sucrose overnight), submerged in OCT (Tissue-Tek, Sakura) and flash frozen. 14 µm thick tissue sections were collected on SuperFrost Plus slides (Fisher). To obtain a cochlear surface preparation, PFA fixed cochlear tissue was dissected in 1X PBS to remove the cochlear capsule, the cochlear roof and the innervating spiral ganglion.

### Immuno-staining

Cochlear tissue was washed three times with 1X PBS 5–10 minutes each and blocked with 1X PBS containing 10% Normal Donkey Serum (Sigma) and 0.5% TritonX100 (Sigma) for 30 minutes. Immuno-staining was performed according to the manufacture’s specifications. Primary antibodies: rabbit anti-MyosinVIIa (1:500, Proteus #25-6790), goat anti-SOX2 (1:500, Santa Cruz #sc-17320), rabbit anti-S100 (1:500, Abcam #ab868). Alex Fluor (488, 546, and 633) labeled secondary antibodies were used to visualize staining (1:1000, Life Technologies). Stereocilia were visualized with fluorescently labeled phalloidin (1:500, Life Technologies). To detect dying cells, an *in situ* cell death detection fluorescein kit was used according to manufactures instructions (Roche).

### *In situ* hybridization (ISH)

300–500 bp fragments of coding sequence of murine *Lfng*, *Shc3, Dkk3, Daam2* were cloned into pGem®-T easy (Promega) and were used as templates to synthesize digoxigenin-labeled antisense RNA probes according to the manufacturer’s specifications (Roche). Probe hybridization and washes were performed as previously described^88^. Bound probe was detected with anti-DIG-AP (alkaline phosphatase conjugated) antibody (Roche) followed by a color reaction using the AP substrate BM-Purple (Roche).

### Quantification of HCs and SCs

Cell counts were performed in cochlear sections or cochlear whole mount preparations immuno-stained for HC marker MYO7A and SC marker SOX2. HC and SC subtypes were identified by their location within the sensory epithelium. For cochlear whole mounts a minimum of two low power confocal z-stacks through the HC layer and the corresponding SC layer were taken in the cochlear base, mid and apex. The length of the imaged segment as well as the total length of the cochlear sensory epithelia was analyzed using Image J (http://imagej.nih.gov/ij). The number of HCs and SCs was manually counted in Photoshop CS5. A minimum of three cochleae obtained from different animals was analyzed for each genotype.

### Statistical analysis

Values are presented as mean ± S.E.M, n = animals per group. All results were confirmed by at least two separate experiments. Two-tailed Student’s t tests were used to determine confidence interval. P-values ≤ 0.05 were considered significant. P-values > 0.05 were considered not significant.

## Additional Information

**How to cite this article**: Campbell, D. P. *et al*. Canonical Notch signaling plays an instructive role in auditory supporting cell development. *Sci. Rep*. **6**, 19484; doi: 10.1038/srep19484 (2016).

## Supplementary Material

Supplementary Information

## Figures and Tables

**Table 1:** List of top-ranked DAPT down-regulated genes.

SD DAPT vs Control Log2(FC)	Gene Symbol	Gene Accession ID	P-value DAPT vs Control	Mean DAPT	Mean Control	Ratio DAPT vs Control	qPCR Ratio DAPT vs Control
≤ − 6σ	Hey1*	NM_010423	0.007	9.23	10.52	0.41	0.18
≤ − 6σ	Jag1*	NM_013822	0.002	10.06	11.30	0.42	ND
≤ − 6σ	Slitrk6	NM_175499	0.002	10.83	12.01	0.44	0.17
≤ − 6σ	Heyl*	NM_013905	0.020	8.08	9.25	0.44	ND
≤ − 6σ	Slc22a3	NM_011395	0.012	7.76	8.91	0.45	0.13
≤ − 6σ	Igfbp3	NM_008343	0.004	9.23	10.36	0.46	0.33
−6σ	Fabp7	NM_021272	0.054	8.56	9.53	0.51	0.34
−6σ	B3galt2	NM_020025	0.017	8.73	9.67	0.52	0.49
−6σ	Fam159b	NM_029984	0.012	5.99	6.85	0.55	ND
−6σ	Sox2*	NM_011443	0.021	11.22	12.07	0.55	ND
−6σ	Shc3	NM_009167	0.020	9.56	10.38	0.57	0.45
−6σ	Hey2*	NM_013904	0.016	9.38	10.16	0.58	ND
−6σ	Hes1*	NM_008235	0.009	8.49	9.25	0.59	ND
−6σ	Trh	NM_009426	0.012	7.38	8.14	0.59	0.13
−6σ	Gucy1b3	NM_017469	0.007	7.59	8.31	0.61	0.56
−6σ	Colgalt2	NM_177756	0.016	9.09	9.76	0.63	0.41
−6σ	Inhba	NM_008380	0.003	9.32	9.98	0.63	0.35
−6σ	Crhbp^#^	NM_198408	0.001	6.52	7.16	0.64	0.78
−6σ	Gucy1a3	NM_021896	0.034	8.49	9.11	0.65	0.49
−6σ	Gpr126	NM_001002268	0.051	8.01	8.63	0.65	0.52
−6σ	Tmem211	NM_001033428	0.001	7.76	8.38	0.65	0.32
−6σ	Gp5	NM_008148	0.032	5.61	6.21	0.66	ND
−6σ	Dkk3	NM_015814	0.030	8.42	9.02	0.66	0.45
−6σ	Xist^#^	NR_001463	0.046	11.40	11.99	0.67	7.04
−6σ	Trhr	NM_013696	0.034	6.68	7.25	0.67	0.33
−6σ	Ntf3	NM_001164034	0.042	8.99	9.57	0.67	0.31
−6σ	Cybrd1	NM_028593	0.021	9.55	10.12	0.67	0.38
−6σ	Moxd1	NM_021509	0.050	10.50	11.06	0.68	0.62
−6σ	Mmd2	NM_175217	0.012	8.26	8.82	0.68	0.52
−6σ	Hes5*	NM_010419	0.008	9.12	9.68	0.68	ND
−6σ	Lacc1	BC116748	0.004	8.89	9.43	0.69	ND
−6σ	Fgf20*	NM_030610	0.029	8.38	8.92	0.69	ND
−6σ	Lfng	NM_008494	0.003	9.23	9.76	0.69	0.38
−6σ	Nrarp	NM_025980	0.012	9.20	9.73	0.69	0.27
−6σ	Cyp26b1	NM_175475	0.054	8.23	8.76	0.70	0.29
−6σ	C030013G03Rik	AK021075	0.021	5.07	5.60	0.70	ND

Genes are ranked based on fold change. Shown are mean normalized log2 transcript signal values for DAPT and control. Note only transcripts with more than 12 probes assigned to them are listed. ^*^indicates known Notch target gene; ^#^indicates that gene failed qPCR validation. Abbreviations: FC, fold change, ND, not determined; SD, standard deviation.
