# Supplementary material for: Canonical Notch signaling plays an instructive role in auditory supporting cell development
Source: Sci Rep. 2016 Jan 20;6:19484. doi: 10.1038/srep19484 (PMC4726253; doi:10.1038/srep19484)
Supplement: Supplementary Information [file srep19484-s1.pdf]

Title: Canonical Notch signaling plays an instructive role in auditory supporting cell development

Author list: Dean Paul Campbell, Elena Chrysostomou and Angelika Doetzlhofer

Supplementary Fig. S1

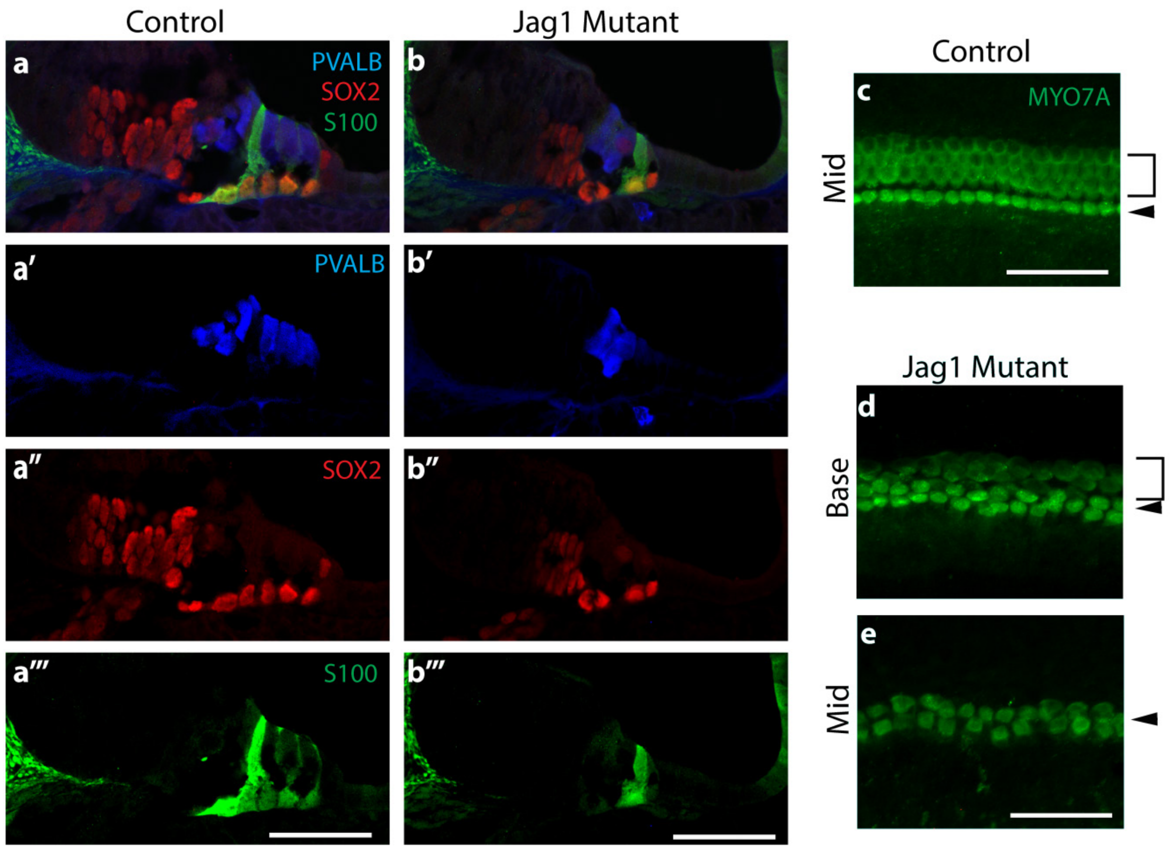

Ablation of *Jag1* at the onset of cochlear differentiation causes severe defects in Deiters' cell and outer HC development. (a- b''') Confocal images of control (*Emx2*$^{+/+}$; *Jag1*$^{fx/fx}$) (a-a''') and *Jag1* mutant (*Emx2*$^{Cre/+}$; *Jag1*$^{\Delta/\Delta}$) (b-b''') cochlear cross-sections stage P2. Parvalbumin (blue) immuno-staining labels HCs, SOX2 (red) immuno-staining labels GER and SCs and S100 (green) immuno-staining labels Deiters' cells and pillar cells. Note outer HCs and their surrounding outer SCs (Deiters' cells and outer pillar cells) are missing in the *Jag1* mutant cochlea. **(c-e)** Confocal images of control (*Emx2*$^{+/+}$; *Jag1*$^{fx/fx}$) (c) and *Jag1* mutant (*Emx2*$^{Cre/+}$; *Jag1*$^{\Delta/\Delta}$) (e, d) cochlear whole mounts stage P2, stained for HC specific protein MYO7A (green).

In *Jag1* mutant sensory epithelia inner HCs (arrowhead) are overproduced as shown here for mid-base (d) and mid (e), whereas outer HCs (bracket) are largely absent. Scale bars for all panels, 50μm.

Supplementary Table S1: List of top ranked DAPT upregulated genes.

Genes are ranked based on fold change (ratio). Shown are mean normalized log2 transcript signal values for DAPT and control. Note only transcript with more than 12 probes assigned to them and with a p-value less or equal to 0.05 are listed. Abbreviation: FC, fold change SD, standard deviation.

SD DAPT vs Control Log2(FC)	*Gene Symbol*	*Gene Accession ID*	*P-value DAPT vs Control*	*Mean DAPT*	*Mean Control*	*Ratio DAPT vs Control*
> +6σ	Tmem173	NM_028261	0.001	10.31	9.07	2.37
> +6σ	Atoh1	NM_007500	0.003	11.54	10.32	2.32
> +6σ	Acbd7	NM_030063	0.004	7.59	6.46	2.19
> +6σ	Pou4f3	NM_138945	0.004	11.08	9.97	2.16
> +6σ	Gm6537	NM_001195091	0.008	10.43	9.33	2.16
> +6σ	Grp	NM_175012	0.004	9.26	8.17	2.12
> +6σ	Tmem255b	NM_001143671	0.030	10.40	9.33	2.11
+6σ	Nhlh1	NM_010916	0.004	8.78	7.74	2.06
+6σ	Calb2	NM_007586	0.002	8.32	7.32	2.00
+6σ	Dlk2	NM_207666	0.002	10.11	9.12	1.99
+6σ	Ush2a	NM_021408	0.037	8.28	7.30	1.97
+6σ	Chrna10	NM_001081424	0.016	8.39	7.43	1.94
+6σ	Ptprq	NM_001081432	0.038	9.08	8.17	1.88
+6σ	Kcna10	NM_001081140	0.016	6.64	5.76	1.85
+6σ	Dll3	NM_007866	0.009	9.09	8.23	1.82
+6σ	Rasd2	NM_029182	0.002	10.07	9.22	1.79
+6σ	Scn11a	NM_011887	0.010	7.88	7.04	1.79

+6σ	Lhfpl5	NM_026571	0.006	8.58	7.78	1.73
+6σ	Rbm24	NM_001081425	0.005	8.26	7.47	1.73
+6σ	Foxj1	NM_008240	0.026	9.30	8.51	1.73
+6σ	Mfng	NM_008595	0.002	9.83	9.04	1.73
+6σ	Steap4	NM_054098	0.046	6.90	6.13	1.70
+6σ	Gad2	NM_008078	0.004	7.84	7.12	1.65
+6σ	Serpine3	AK053602	0.007	7.19	6.47	1.64
+6σ	Tmprss7	NM_172455	0.001	7.23	6.53	1.62
+6σ	Rtn4rl2	NM_199223	0.007	9.92	9.24	1.60
+6σ	Lhx3	NM_001039653	0.040	8.95	8.27	1.60
+6σ	Dysfip1	NM_026814	0.048	7.53	6.86	1.59
+6σ	Mgat5b	NM_172948	0.009	9.36	8.71	1.58
+6σ	Gm88	BC147714	0.001	8.69	8.04	1.57
+6σ	Slc26a5	NM_030727	0.036	6.78	6.14	1.56
+6σ	Otof	NM_031875	0.027	8.89	8.26	1.56
+6σ	Oacyl	NM_177028	0.003	8.30	7.66	1.56
+6σ	Gimap4	NM_174990	0.041	7.19	6.55	1.55
+6σ	Gm2694	NR_033430	0.044	7.53	6.90	1.55
+6σ	Bsnd	NM_080458	0.046	9.02	8.40	1.54
+6σ	Thsd7b	NM_172485	0.017	7.87	7.25	1.54
+6σ	Aqp1	NM_007472	0.040	9.56	8.95	1.52
+6σ	Jag2	NM_010588	0.004	10.15	9.55	1.52
+6σ	Dll1	NM_007865	0.000	8.01	7.41	1.52
+6σ	Ankrd22	NM_024204	0.020	8.76	8.17	1.50
+6σ	Wfikkn2	NM_181819	0.008	9.47	8.88	1.50
+6σ	Tesc	NM_021344	0.011	8.09	7.52	1.48
+6σ	Slc52a3	NM_027172	0.017	9.86	9.30	1.48
+6σ	Gm11992	NM_001037928	0.024	7.35	6.79	1.48
+6σ	Apln	NM_013912	0.048	8.78	8.22	1.47
+6σ	Mep1a	NM_008585	0.008	6.03	5.47	1.47
+6σ	Srrm4	NM_026886	0.014	7.64	7.09	1.47

+6σ	Gm8075	XR_105495	0.004	5.75	5.20	1.47
+6σ	Casz1	NM_027195	0.009	8.36	7.82	1.46
+6σ	Sstr2	NM_001042606	0.040	8.99	8.45	1.45
+6σ	Pde2a	NM_001143848	0.009	8.62	8.08	1.45
+6σ	Cbln1	NM_019626	0.004	7.58	7.05	1.45
+6σ	Fam183b	NM_029283	0.011	6.61	6.07	1.45

Supplementary Table S2: Gene ontology analysis of DAPT-downregulated genes.

Listed are significantly enriched biological processes as well as the number, percentage and name of DAPT-down-regulated genes involved in the term. Note the list of DAPT down-regulated genes (FC -1.215, p value ≤0.07) contained 107 genes of which 74.8% had functional annotation in the analyzed biological process category (GOTERM_BP_FAT).

Term	Count	%	P-Value	Gene symbol	Fold Enrichment
GO:0001709~cell fate determination	5	4.7	1.68E-05	HES1, NTF3, HES5, CYP26B1, PROX1	31.5
GO:0042490~mechanoreceptor differentiation	5	4.7	5.41E-05	HES1, NTF3, HES5, JAG1, FGF20	23.6
GO:0045165~cell fate commitment	7	6.5	2.10E-04	HES1, NTF3, HES5, SOX2, CYP26B1, PROX1, FGF3	8.1
GO:0060284~regulation of cell development	7	6.5	3.21E-04	HES1, NTF3, HES5, SOX2, PROX1, IGFBP3, WNT7A	7.5
GO:0060113~inner ear receptor cell differentiation	4	3.7	6.86E-04	HES1, HES5, JAG1, FGF20	22.6
GO:0008593~regulation of Notch signaling pathway	3	2.8	9.14E-04	SOX2, HEY2, JAG1	63.7
GO:0051094~positive regulation of developmental process	7	6.5	1.52E-03	NTF3, SOX2, HEY2, JAG1, PROX1, IGFBP3, WNT7A	5.6
GO:0048839~inner ear development	5	4.7	1.70E-03	HES1, HES5, SOX2, JAG1, FGF20	9.7
GO:0030182~neuron differentiation	9	8.4	2.18E-03	HES1, NTF3, HES5, PTPRZ1, SOX2, JAG1, SLITRK6, FGF20, WNT7A	3.8
GO:0007219~Notch signaling pathway	4	3.7	3.79E-03	HEY1, HEYL, HEY2, JAG1	12.6
GO:0046068~cGMP metabolic process	3	2.8	3.80E-03	PDE5A, GUCY1A3, GUCY1B3	31.8
GO:0007267~cell-cell signaling	7	6.5	6.78E-03	NTF3, SOX2, SLC22A3, SHC3, WNT7A, FGF3, LIN7A	4.1

GO:0050767~regulation of neurogenesis	5	4.7	7.29E-03	HES1, NTF3, HES5, SOX2, WNT7A	6.4
GO:0006836~neurotransmitter transport	4	3.7	1.13E-02	SLC6A2, SLC22A3, WNT7A, LIN7A	8.5
GO:0045665~negative regulation of neuron differentiation	3	2.8	1.66E-02	HES1, HES5, SOX2	15.0
GO:0007423~sensory organ development	6	5.6	1.69E-02	HES1, HES5, SOX2, JAG1, FGF20, PROX1	4.0
GO:0042668~auditory receptor cell fate determination	2	1.9	1.73E-02	HES1, HES5	113.2
GO:0007268~synaptic transmission	5	4.7	2.00E-02	NTF3, SLC22A3, SHC3, WNT7A, LIN7A	4.8
GO:0008284~positive regulation of cell proliferation	6	5.6	2.48E-02	HES1, SOX2, HEY2, FGF20, PROX1, FGF3	3.6
GO:0007389~pattern specification process	6	5.6	2.48E-02	HES1, FLT1, CYP26B1, MSGN1, WNT7A, LFNG	3.6
GO:0048514~blood vessel morphogenesis	5	4.7	2.82E-02	FLT1, HEY1, HEY2, PROX1, TNFAIP2	4.3
GO:0000904~cell morphogenesis involved in differentiation	5	4.7	3.49E-02	HES1, NTF3, PTPRZ1, SLITRK6, PROX1	4.0
GO:0007166~cell surface receptor linked signal transduction	22	20.6	4.49E-02	GPR126, FLT1, GDF6, TRHR, F2RL1, JAG1, FGF20, MRGPRH, FZD4, HES1, DKK3, HEY1, HES5, HEY2, HEYL, RGS5, PDGFRB, OLFR1162, WNT7A, ADAMTS5, FGF3, CCRL1	1.5

Supplementary Table S3: The HC to SC ratio is altered in N1ICD mutants. HCs and SCs were quantified in cochlear sections obtained from three *Emx2*$^{Cre/+}$*; ROSA26*$^{N1ICD/+}$ (N1ICD) embryos and three corresponding wild type (control) littermates. Adjacent sections were immuno-stained for MYO7A and SOX2 and S100A1 and SOX2. HCs were identified by MYO7A staining and their medial lateral position within the sensory epithelium was used to classify HCs as inner or outer HCs; outer SCs (SC-like cells) were identified by their expression of SOX2 and S100A1 in adjacent sections.

**N1ICD**	Number of sections	Mean number of IHC	Mean number of OHC	Mean number of HC	Mean number of SC	HC/ SC	SC/ HC	SC/ OHC
R1	7	1.00	0.57	1.57	7.86	0.20	5.00	13.75
R2	6	1.00	0.83	1.83	7.00	0.26	3.82	8.40
NE8	6	0.83	1.33	2.17	7.33	0.30	3.38	5.50
	**Mean**	**0.94**	**0.91**	**1.86**	**7.40**	**0.25**	**3.97**	**8.10**
	SEM	0.06	0.22	0.17	0.25	0.03	0.48	2.42

**Control**	Number of sections	Mean number of IHC	Mean number of OHC	Mean number of HC	Mean number of SC	HC/ SC	SC/ HC	SC/OHC
NE2	6	1.00	3.00	4.00	5.17	0.78	1.29	1.72
R3	5	1.00	3.00	4.00	5.20	0.77	1.30	1.73
R4	5	1.00	3.00	4.00	5.00	0.80	1.25	1.67
	**Mean**	**1.00**	**3.00**	**4.00**	**5.12**	**0.78**	**1.28**	**1.71**
	SEM	0.00	0.00	0.00	0.05	0.01	0.01	0.02

Supplementary Table S4: List of qPCR primers used in this study

**Gene**	**Forward Primer Sequence**	**Reverse Primer Sequence**
Abcc9	TGG AGG TCA GGA CGG ACT ATC T	GCC ACT AAT GGA TGC AAT GGA
Atoh1	ATG CAC GGG CTG AAC CA	TCG TTG TTG AAG GAC GGG ATA
B3galt2	GCA CCG AAC AGA AAC AAA GAC A	TAG CGC TCA CTT GGG TAA AGG
Chst1	GGCTACAAGATGGCCAACTCA	ACGCTCCTCCACTAGGCTGAT
Colgalt2	TTC AGT CCA AAT GCC CAG TTC	CCA TGT TGC CAC ACC AGT GT
Crhbp	TGG AGC TGC TGG GAG GAA	CAG GTC TGC TAA GGG CAT CAT C

Cybrd1	AGA CTG CCA TGG ACC TGG AA	CCG GCA TGG ATG GAT TTC
Cyp26b1	TCT GCC CCT TTG CTC TTG	ACA GGG ATC CCC TTC AGC
Daam2	GCA GTG GAA GTG GAG TTG GAA	CAG GGA CGA ACT TGT CAT TGG
Dkk3	TGT GTA CAC TGC TGG CGG CG	GAG CTC TCC CTC CAC GGG CA
Fabp7	GGA AGG TGG CAA AGT GGT GAT	TGG AAA TTG ATC TCT GTG TTC TTG A
Fgfr3	GGG CTC CTT ATT GGA CTC G	GCT CCC CTC GGA ATT CTT T
Gpr126	GCA ACC GGA CCC TGA GAG A	GTC ATG CCA AGC AGG AAG GT
Gucy1a3	TCC CCG CTT CGC TCT TCT	CCA GGT CTC GGT CCA GCA T
Gucy1b3	ATG AAC CTG GAC GAC CTA ACA AG	AGC ATC GTG GAG AGG GAT GT
Hes5	GGC GGT GGA GAT GCT CAG T	GCT GCT CTA TGC TGC TGT TGA
Hey1	CAC TGC AGG AGG GAA AGG TTA T	CCC CAA ACT CCG ATA GTC CAT
Heyl	GCG CAG AGG GAT CAT AGA GAA	TCG CAA TTC AGA AAG GCT ACT G
Igfbp3	AAC CTG CTC CAG GAA ACA TCA GT	GCT TTC CAC ACT CCC AGC AT
Inhba	TCA GGC ACA GCC AGG AAG A	TGA CAG GTC ACT GCC TTC CTT
Jag1	TGT GCA AAC ATC ACT TTC ACC TTT	GCA AAT GTG TTC GGT GGT AAG AC
Lfng	ACT GCA CCA TTG GCT ACA TTG T	GGC CGC TCC GGA TGA
Mmd2	TCC CGG CGC ACA AGA G	CAC AGT TTG CTG CGT GTT CA
Moxd1	TGA CAG CGT TCT GGA CTT TGG	GAA GGC ATC GGG CAT GTT
Nrarp	TCGCTGCTGCAGAACATGAC	CTCCGGCCCGAACGA
Nckap5	TCC AAC TGC CAG ATG AGA ACA CT	GGC ATA TGT ATC GTC CCA CTG A
Ntf3	CCA AGG CAA CAG CAT GGA T	AGC TTG ATG ATG AGG GAA TTG AG
Otog	CCA TCA GCT GCC CTC CAT	GTA CCA CAG AGC CAC CAA CCT T
Pdgfrb	GTC CCA TCT GCC CCT GAA A	CTG TGT AGC TGA GCA CTG GTG AGT
Prox1	CGT TAC GGG AGT TTT TCA ATG C	CCT TGT AAA TGG CCT TCT TCC A
Rgs5	GCC CCT AAA GAG GTG AAC ATT G	GAC GGT TCC ACC AGG TTC TTC
Rpl19	GGT CTG GTT GGA TCC CAA TG	CCC GGG AAT GGA CAG TCA
S100a1	TGG ATG TCC AGA AGG ATG CA	CCG TTT TCA TCC AGT TCC TTC A
Shc3	GAG AAA GCC GCC GAG TAA GAT	GCG AAC TGG AGG TTG CTC TTC
Slc1a3	AGT GCC TAT CCA GTC CAA CGA	GGC CTC TGA CAC GTT GTT GA
Slc22a3	GCT CAT CCT TAT GTT TGC TTG GT	GCG CAT GAC AAG TCC TTG GT
Slc6a14	TCT GTG TGA CTC AGG CTG GAA	CCC ATC CAG CAC AGA AGT GA
Slitrk6	CTT CCA GCT GGG CAT TTC A	TGA TTG GAT CTG ACT CTG TAA AGC A
Sox2	CCA GCG CAT GGA CAG CTA	GCT GCT CCT GCA TCA TGC T
Tmem211	GGA AGG TCT CAG CTG CAA CAC	AAG AGC GCT GAT TGA CAG CAA
Trh	TGA TGG CTC TGG CTT TGA TCT	CAG CAA GGC GCA GGA TTT
Trhr	TCA CCG TCA CCG ATA CGT ATG T	GGC CAA GCA GGT GTC ATC A
Xist	AAG GAA ACC TGA ACA GCG TAA AA	AAT GAG ATG TGT GCA GTA AAT GCA
